# Enhanced early chronic kidney disease prediction using hybrid waterwheel plant algorithm for deep neural network optimization

**DOI:** 10.1038/s41598-025-26382-6

**Published:** 2025-11-27

**Authors:** Doaa Sami Khafaga, Nima Khodadadi, Ehsaneh Khodadadi, Amel Ali Alhussan, Marwa M. Eid, El-Sayed M. El-Kenawy

**Affiliations:** 1 Department of Computer Sciences, College of Computer and Information Sciences, Princess Nourah bint Abdulrahman University, P.O. Box 84428, Riyadh, 11671 Saudi Arabia; 2 Department of Civil and Environmental Engineering, University of California, Berkeley, CA USA; 3 Department of Chemistry and Biochemistry, University of Arkansas, Fayetteville, Fayetteville, AR 72701 USA; 4 Faculty of Artificial Intelligence, Delta University for Science and Technology, Mansoura, 11152 Egypt; 5 Jadara Research Center, Jadara University, Irbid, 21110 Jordan; 6 Department of Communications and Electronics, Delta Higher Institute of Engineering and Technology, Mansoura, 35111 Egypt; 7 Applied Science Research Center, Applied Science Private University, Amman, 11931 Jordan

**Keywords:** Chronic kidney disease prediction, Deep neural networks, Hybrid optimization algorithms, Waterwheel plant algorithm, Grey wolf optimization, Early medical diagnosis, Computational biology and bioinformatics, Engineering, Mathematics and computing, Nephrology

## Abstract

Chronic Kidney Disease (CKD) is a progressive condition primarily caused by diabetes and hypertension, affecting millions worldwide. Early diagnosis remains a clinical challenge since traditional approaches, such as Glomerular Filtration Rate (GFR) estimation and kidney damage indicators, often fail to detect CKD in its initial stages. This study aims to enhance early CKD prediction by developing a deep neural network optimized with a novel hybrid metaheuristic that combines the Waterwheel Plant Algorithm (WWPA) with Grey Wolf Optimization (GWO). Using the UCI CKD dataset, rigorous preprocessing techniques-including data imputation, normalization, and synthetic oversampling-were employed to enhance data quality and mitigate class imbalance. A multilayer perceptron (MLP) regression model was trained and optimized through the WWPA-GWO framework and benchmarked against other optimization algorithms, including PSO, GA, and WOA. Results demonstrated that the standard MLP achieved moderate performance (MSE = 0.00177, RMSE = 0.0420, MAE = 0.0100, $R^2$ = 0.8793), whereas the optimized model achieved significant improvements (MSE = $3.06 \times 10^{-6}$, RMSE = 0.00175, $R^2$ = 0.9730) with reduced computational time (0.0999 s). Statistical validation using ANOVA ($p < 0.0001$) and Wilcoxon signed-rank testing ($p = 0.002$) confirmed the robustness of the approach. These findings highlight the effectiveness of the WWPA-GWO hybrid optimization strategy for deep neural networks, offering a reliable and efficient pathway for early CKD detection. Future work will explore the integration of advanced imputation methods, multi-modal data sources, and federated learning frameworks to enhance the model’s generalizability and clinical utility in diverse healthcare settings.

## Introduction

Chronic kidney disease (CKD) affects approximately 9% of the global population, representing over 674 million individuals, and is increasingly recognized as a leading cause of premature mortality worldwide. Recent estimates project that CKD will become the fifth leading cause of years of life lost (YLL) by 2040, surpassing diseases such as cancer and diabetes. In 2021 alone, CKD was associated with 1.5 million deaths globally, with mortality expected to rise to over 2.2 million by 2040 under current progression trends.

The health system burden of CKD is particularly severe due to the high costs associated with end-stage treatments. Hemodialysis and peritoneal dialysis remain financially burdensome, with annual costs per patient exceeding $50,000 in high-income countries and rapidly rising in middle-income economies. Global kidney replacement therapy (KRT) expenditures are projected to increase from $169.6 billion in 2022 to over $186.6 billion by 2027. This economic impact, compounded by limited access to treatment in resource-poor settings, underscores the need for improved early detection strategies.

The progressive decline in kidney function characterizes CKD over time. It is a growing and emerging cause of death globally, ranking number five by 2040^1^. In high-income countries, the annual increase in healthcare costs due to transplantation and dialysis is 2–3%^2^. Dialysis or kidney transplantation in poor and middle-income countries remains difficult as most patients with renal failure live there^3^. New economies, such as China and India, can be expected to record more renal failure cases than previously observed^4^. More fluids in the blood cannot be easily expelled from the body when the kidneys’ function is gradually impaired, leading to complications such as hypertension, anemia, osteoporosis, and nerve damage. Glomerular filtration rate (GFR) remains the gold standard for measuring kidney health^5^.

Furthermore, the GFR helps doctors in determining the presence of renal disease. Both chronic kidney damage lasting three months and a GFR of less than 60 mL/min/1.73 m^2^ over the same period are needed to diagnose CKD. Five stages are recognized for CKD, and at each stage, the GFR worsens; it can be determined using the GFR^6^, which is the most accurate estimator of renal activity. If the GFR levels are less than 15 mL/min, the patient is in renal failure^6^. Diagnosing CKD is not easy since there are many causes of the disease, and physicians’ experience plays a key role^7^. With the growing complexity of healthcare data and the introduction of new diagnostic techniques, machine learning (ML) offers a reliable and automated way to assist in early detection^8,9^.

ML has emerged as a transformative approach for enhancing disease diagnostics by uncovering complex, non-linear patterns within large and heterogeneous medical datasets. In the context of chronic diseases, ML-based models offer significant advantages in automating diagnostic workflows, reducing reliance on subjective clinical judgment, and increasing early detection accuracy. Recent studies have illustrated the effectiveness of ML algorithms in predicting various disorders, including polycystic ovary syndrome (PCOS), heart disease, thyroid dysfunction, and arrhythmias^10–13^. For example, Kumar et al.^10^ demonstrated that a hybrid logistic regression model enhanced with particle swarm optimization achieved an accuracy of 96.3% in PCOS diagnosis, highlighting the value of optimization-enhanced classifiers. Similarly, Lai et al.^14^ showed that integrating improved Grey Wolf Optimization with artificial neural networks can substantially enhance diagnostic accuracy in skin cancer detection, while Ghafariasl et al.^15^ fine-tuned pre-trained deep networks for superior breast cancer classification performance. These studies further emphasize the importance of hybrid and optimization-based deep learning strategies for improving medical prediction tasks.

Despite these advancements, traditional ML techniques often treat feature selection and hyperparameter tuning as independent processes, leading to suboptimal diagnostic performance, especially in high-dimensional biomedical data. A recent systematic review emphasized that integrating these two processes through hybrid optimization frameworks can substantially improve diagnostic accuracy, computational efficiency, and model interpretability^16^. These frameworks employ metaheuristic algorithms in combination with classifiers like Support Vector Machines (SVM), Decision Trees (DT), and Random Forests (RF), yielding 12–15% gains in classification accuracy across diseases such as cardiovascular conditions, diabetes, and various cancers. The review also highlighted the growing importance of cross-validation, federated learning, and explainable AI (XAI) in building clinically actionable and generalizable models.

In the specific case of CKD, ML techniques such as Random Forest^17^, Fuzzy C-Means^18^, Naive Bayes^19^, and SVMs^20,21^ have been widely applied. However, these models are frequently limited by their inability to handle the intricate, high-dimensional nature of clinical data without significant feature engineering or tuning. Moreover, they often face issues of overfitting, convergence to local optima, and high computational costs. Consequently, there is an emerging research need to develop hybrid optimization frameworks that can jointly optimize feature subsets and classifier hyperparameters, thereby producing more robust and efficient diagnostic models tailored for early CKD detection.

Artificial Neural Networks (ANNs), or more specifically, deep neural networks (DNNs), are increasingly preferred for medical diagnostics because they can directly learn non-linear relationships within datasets and automatically extract relevant features. Nevertheless, their performance is highly sensitive to hyperparameter settings, which can be enhanced through sophisticated optimization strategies. Common optimization methods often suffer from premature convergence and entrapment in local optima, which limits their capacity to generalize.

To overcome these challenges, this study proposes a hybrid Waterwheel Plant Algorithm–Grey Wolf Optimization (WWPA–GWO) framework that leverages the complementary strengths of both methods. The WWPA contributes robust *exploration* by dynamically diversifying the search space and preventing early stagnation, while GWO offers efficient *exploitation* by refining solutions around promising regions through its hierarchical leadership mechanism. This synergistic combination ensures a more balanced and adaptive optimization process, enhancing the convergence speed, stability, and predictive performance of the DNN for early CKD prediction.

In this study, the primary computational problem addressed is the inefficient and suboptimal hyperparameter optimization of DNNs for early CKD prediction. Conventional techniques such as grid search, and random search incur high computational costs or converge prematurely to local optima, resulting in limited model performance. The proposed WWPA–GWO hybrid algorithm effectively integrates biological inspiration and swarm intelligence to optimize DNN hyperparameters, yielding higher diagnostic accuracy, lower computational cost, and improved robustness.

## Literature review

Predictive modeling for osteoporosis in patients with CKD has shown promising results using machine learning approaches. Research by^22^ demonstrated that Random Forest algorithms achieved exceptional predictive capabilities, especially among female patients who exhibited a 17.57% disease prevalence. The female-specific model identified critical predictors, including body weight, hormone replacement therapy usage, patient age, ethnic background, and erythrocyte counts, culminating in a clinically validated predictive instrument.

The absence of reliable diagnostic markers for CKD presents a significant challenge in contemporary healthcare. Research findings from^23^ revealed that sophisticated bioinformatics methodologies successfully identified four key tubular damage biomarkers-DUSP1, GADD45A, TSC22D3, and ZFAND5-which demonstrate crucial roles in immune regulation and inflammatory cascades, showing strong correlations with established parameters such as Glomerular Filtration Rate and creatinine levels.

With approximately 697.5 million individuals affected globally, CKD represents a substantial health burden. The investigation by^24^ suggests that conventional analytical approaches may inadequately capture the intricate, non-linear connections between environmental exposures and CKD development. Advanced interpretable machine learning techniques revealed meaningful correlations, particularly identifying urinary volatile organic compound metabolites, such as N-Acetyl-S-(3,4-dihydroxybutyl)-L-cysteine (DHBMA), as crucial disease predictors, potentially informing targeted preventive interventions.

Patients diagnosed with CKD experience elevated risks for medication-related complications due to multiple comorbidities and complex pharmaceutical regimens. The study by^25^ successfully employed machine learning methodology, particularly random forest algorithms, to identify high-risk CKD patients for medication therapy problems within primary care environments, utilizing standard clinical parameters including diabetic status, glycated hemoglobin levels, and blood pressure measurements.

The global burden of CKD is compounded by its frequent association with dilated cardiomyopathy, substantially elevating cardiovascular risk profiles. Research conducted by^26^ addressed the diagnostic challenges in detecting dilated cardiomyopathy among CKD patients, building upon established evidence linking renal impairment to subsequent cardiac pathology.

Multiple patient characteristics, including age, hemoglobin levels, educational background, and social engagement, have been identified as important determinants of cognitive decline in populations with CKD. The research by^27^ demonstrated that neural network models achieved superior predictive capabilities for this complication, with subsequent feature analysis revealing age, educational attainment, and hemoglobin concentration as primary influential variables.

Coronary artery disease prevalence among CKD patients substantially elevates their cardiovascular morbidity and mortality risks. The investigation by^28^ established that inflammatory pathways play central roles in CAD development within CKD populations, leading to the identification of promising biomarkers, including glutamate cysteine ligase modifier subunit (GCLM) and nuclear protein 1 (NUPR1), for enhanced diagnostic and therapeutic applications.

Contemporary machine learning approaches are increasingly incorporating multi-biomarker strategies for disease detection, although the economic implications are often inadequately considered. The analysis by^29^ revealed that expanding model features can increase procedural expenses by nearly threefold while providing minimal accuracy improvements, demonstrating substantial disparities between classifier effectiveness and associated costs. These findings suggest that minor performance enhancements may not justify significantly higher expenditures, underscoring the need for cost-benefit analyses in the selection of clinical models.

Social determinants have a significant influence on the development and progression of CKD. The study by^30^ demonstrated that incorporating social determinants into machine learning frameworks, particularly random forest models, substantially improved the accuracy of CKD risk prediction among Type 2 diabetic patients, achieving an impressive area under the receiver operating characteristic curve of 0.89.

Calcification prevalence remains elevated in CKD populations, with abdominal aortic calcification serving as a powerful predictor of cardiac complications. The research by^31^ utilized machine learning models to identify key determinants of calcification across both CKD and non-CKD cohorts. Results indicated that age, smoking history, and estimated glomerular filtration rate consistently emerged as primary influential factors in both groups. Additionally, while glucose levels and albumin-to-creatinine ratios represented shared risk elements, specific inflammatory markers, including monocyte-to-lymphocyte and neutrophil-to-lymphocyte ratios, demonstrated particular importance for calcification progression in CKD patients.

Early-stage CKD detection in diabetic populations remains challenging due to subtle clinical presentations. The study by^32^ addressed these diagnostic difficulties through attention-based deep learning architectures that demonstrated exceptional accuracy in CKD stage classification, highlighting serum creatinine and cystatin C as pivotal classification parameters.

Risk stratification for one-year CKD progression in patients with type 2 diabetes mellitus has been investigated using diverse machine learning methodologies. The analysis by^33^ encompassed ten different algorithmic approaches applied to extensive patient datasets, with XGBoost demonstrating optimal predictive performance for kidney function deterioration and subsequent integration into a clinician-friendly web-based platform.

CKD mineral bone disorder commonly complicates advanced and end-stage kidney disease, significantly increasing fracture and osteoporosis susceptibility. The research by^34^ developed machine learning predictive models, with artificial neural networks showing exceptional accuracy in osteoporosis risk identification among these patient populations, demonstrating substantial clinical screening potential.

Sarcopenia represents a frequent complication in CKD patients, adversely affecting clinical outcomes. The findings by^35^ established that machine learning-based predictive models identified advanced age, waist circumference, low-density lipoprotein cholesterol, high-density lipoprotein cholesterol, triglyceride levels, and diastolic blood pressure as significant determinants of sarcopenia risk in this population.

CKD patients constitute the primary at-risk population for post-contrast acute kidney injury, yet specialized predictive instruments remain limited. The study by^36^ demonstrated that explainable deep neural network models represent valuable tools for predicting this complication in patients undergoing coronary procedures, exhibiting superior performance compared to conventional scoring systems while enabling essential clinical risk assessment.

Feline CKD ranks among the most prevalent veterinary conditions and represents a leading cause of death in cats exceeding five years of age. The investigation by^37^ employed metabolomics methodologies to identify early disease indicators through comparisons between healthy cats and those with early-stage disease. Research identified the serum-to-urine 3-hydroxykynurenine ratio as a significant individual biomarker, while machine learning models incorporating metabolites such as creatinine, symmetric dimethylarginine, and aconitic acid improved diagnostic precision. This sophisticated modeling approach enabled diagnosis approximately six months earlier than conventional techniques, demonstrating pathways toward enhanced diagnostics and timelier disease intervention.

CKD represents a widespread health condition requiring individualized treatment approaches across its five progressive stages. The work by^38^ demonstrated that machine learning and generative artificial intelligence technologies show significant promise for forecasting disease progression using patient information. However, current predictive models encounter constraints regarding generalizability, interpretability, and computational requirements.

Significant correlations have been documented between CKD and exposure to principal xenoestrogens, encompassing phthalates, parabens, and phenolic compounds. The findings by^39^ utilized interpretable machine learning models to demonstrate the substantial predictive value of specific urinary xenoestrogen metabolites, particularly methyl paraben, mono-(carboxynonyl) phthalate, and triclosan, for identifying individuals at risk.

CKD represents a worldwide health challenge that frequently remains undiagnosed due to its initially subtle clinical manifestations, contributing to rising morbidity and mortality rates. The analysis by^40^ implemented an optimized machine learning framework across more than 39,000 research abstracts to identify 68 comorbidities spanning 15 disease categories that influence the development or progression of CKD, thereby advancing the comprehension of prognostic factors.

Patients with concurrent diabetes mellitus and CKD experience elevated cardiovascular event risks, with conventional prediction methodologies demonstrating inadequate performance. The research by^41^ showed that machine learning techniques, particularly Light Gradient Boosting Machine models, can achieve satisfactory predictive capabilities by leveraging key variables including estimated glomerular filtration rate, patient age, and triglyceride glucose index. The methodology and key findings of all the literature reviewed are summarized in Table 1.

**Table 1:** Comparing the recent work related to heart disease.

No.	Main focus	Methodology	Key findings
Ref^22^	Predicting osteoporosis risk in male and female CKD patients using clinical and survey data.	Utilized machine learning models on National Health and Nutrition Examination Survey (NHANES) data, with feature selection via LASSO for sex-specific models.	Successfully developed and validated sex-specific models for predicting osteoporosis in CKD patients, identifying key risk factors for each gender.
Ref^23^	Discovering novel biomarkers from renal tubules for the early clinical detection of CKD.	Combined transcriptomic analysis of renal tubule samples from CKD patients and healthy controls with machine learning algorithms.	Identified a set of tubular injury biomarkers that can aid in early CKD diagnosis and provide insights into its pathophysiology.
Ref^24^	Exploring the non-linear association between volatile organic compound (VOC) exposure and CKD risk.	Applied explainable machine learning methods to analyze urinary VOC metabolite data from a large population survey.	Established a significant association between specific urinary VOCs and CKD risk, demonstrating the value of ML in uncovering complex environmental risk factors.
Ref^25^	Developing a predictive model to identify CKD patients at high risk for medication therapy problems (MTPs).	Built a machine learning model using baseline data from the Kidney Coordinated Health Management Partnership (Kidney CHAMP) trial.	Created a predictive tool to effectively identify high-risk CKD patients for MTPs, enabling targeted interventions in primary care.
Ref^26^	Identifying diagnostic biomarkers for dilated cardiomyopathy (DCM) in patients with CKD.	Integrated bioinformatics analysis of gene expression datasets with machine learning to find common biomarkers for CKD and DCM.	Discovered key shared biomarkers, providing a molecular basis for the early diagnosis of DCM in CKD patients and revealing potential pathogenic links.
Ref^27^	Creating a risk prediction model for cognitive impairment (CI) in the CKD patient population.	Developed and validated a prediction model using machine learning algorithms on clinical data from CKD patients.	Produced an accurate ML model for predicting CI risk in CKD patients, facilitating early screening and intervention to improve quality of life.
Ref^28^	Identifying inflammation-related gene markers that link CKD with coronary artery disease (CAD).	Employed comprehensive bioinformatics analysis and machine learning to analyze gene datasets for common inflammatory markers.	Identified key inflammatory response genes as potential biomarkers for early diagnosis and management of CAD in CKD patients.
Ref^29^	Balancing diagnostic accuracy and economic cost in machine learning models for detecting medial vascular calcification (mVC) in CKD.	Developed ML models integrating multiple biomarkers and conducted a cost-effectiveness analysis for mVC detection.	Demonstrated that cost-effective ML models can be developed for mVC detection, highlighting the importance of balancing clinical accuracy with economic feasibility.
Ref^30^	Predicting CKD in type 2 diabetes patients by incorporating social determinants of health (SDOH).	Developed explainable machine learning models that integrate clinical data with SDOH factors from patient records.	Found that including SDOH factors significantly improved the model’s performance in predicting CKD among diabetic patients.
Ref^31^	Identifying key risk factors for abdominal aortic calcification (AAC) in both CKD and non-CKD populations.	Applied interpretable machine learning methods to NHANES data to compare AAC predictors between CKD and non-CKD groups.	Identified distinct and shared key factors for AAC, suggesting different calcification pathways between the two populations.
Ref^32^	Developing a deep learning model for the early and accurate detection of CKD stages in diabetes patients.	Employed a TabNet deep learning approach on patient data to classify different stages of CKD.	The TabNet model achieved high accuracy in detecting early stages of CKD, offering a powerful tool for timely intervention in diabetic patients.
Ref^33^	Predicting 1-year kidney function progression in patients with type 2 diabetes mellitus (T2DM) and CKD.	Developed and validated various machine learning algorithms on a large retrospective cohort of patients with T2DM and CKD.	Created a robust ML model for predicting 1-year CKD progression, enabling proactive management for high-risk T2DM patients.
Ref^34^	Developing ML models to predict osteoporosis risk specifically in patients with advanced CKD and end-stage kidney disease (ESKD).	Retrospectively analyzed a de-identified osteoporosis dataset using multiple machine learning algorithms.	Successfully developed predictive models to identify patients with advanced CKD (stages 3-5) and ESKD at high risk for osteoporosis.
Ref^35^	Creating a predictive model for sarcopenia risk in individuals with CKD.	Utilized data from the China Health and Retirement Longitudinal Study (CHARLS) to develop both a nomogram and a machine learning model.	Developed and validated an effective predictive tool for sarcopenia risk in CKD patients, aiding early identification and intervention.
Ref^36^	Predicting post-contrast acute kidney injury (PC-AKI) in CKD patients after coronary procedures.	Developed and validated a deep learning model using a cohort of adult CKD patients undergoing coronary angiography or intervention.	The deep learning model accurately predicted PC-AKI risk, providing a specialized tool for pre-procedural risk stratification in this vulnerable population.
Ref^37^	Identifying early metabolic biomarkers for feline CKD using machine learning.	Measured metabolites like 3-hydroxykynurenine in feline samples and applied machine learning to differentiate early CKD from healthy states.	Identified 3-hydroxykynurenine as a promising early biomarker, with ML models effectively diagnosing feline CKD before traditional tests.
Ref^38^	Developing a generalizable and interpretable clinical decision support system for staging CKD.	Utilized Machine Learning and Generative AI to build a system for predicting CKD stages, focusing on overcoming limitations of prior models.	Created an ML-based system for CKD staging that enhances generalizability and interpretability for personalized treatment planning.
Ref^39^	Investigating the impact of urinary xenoestrogen exposure on CKD risk in adults.	Developed an interpretable machine learning model (Random Forest, XGBoost) using NHANES data to predict CKD based on environmental exposures.	Confirmed a strong association between urinary xenoestrogens (phthalates, parabens, phenols) and CKD, identifying key environmental risk factors.
Ref^40^	Systematically extracting CKD comorbidity information from scientific literature abstracts.	Developed a novel machine learning methodology, likely using Natural Language Processing, to automatically extract comorbidity data from text.	Presented an effective ML-based method for automatically extracting CKD comorbidity data from literature, aiding in risk-group identification.
Ref^41^	Predicting major adverse cardiac events (MACE) in patients with both diabetes (DM) and CKD.	Applied a machine learning model to a routine care dataset (Silesia Diabetes-Heart Project) to predict cardiovascular events.	The ML model demonstrated superior performance over traditional risk prediction methods for assessing cardiovascular risk in the high-risk DM and CKD population.

Based on these limitations, this study presents a hybrid deep learning framework that combines the Waterwheel Plant Algorithm (WWPA) and Grey Wolf Optimization (GWO) to optimize deep neural network parameters. Our model aims to fill the gap by enhancing prediction accuracy, reducing computational time, and statistically validating the model’s superiority over traditional methods.

From the reviewed literature, it is evident that while machine learning techniques such as Random Forest, SVM, Naive Bayes, and hybrid models have been applied to CKD prediction and related conditions, several critical gaps remain unaddressed. First, most existing studies focus primarily on conventional ML classifiers without integrating advanced deep learning architectures capable of capturing complex, non-linear patterns in high-dimensional clinical data. Second, even when metaheuristic optimization methods are employed, they are often applied in isolation to either feature selection or hyperparameter tuning, rather than jointly optimizing model parameters in a unified framework. Third, few studies systematically evaluate the trade-off between predictive performance and computational efficiency, and even fewer validate their findings with rigorous statistical tests such as Analysis of Variance (ANOVA) or Wilcoxon signed-rank tests. This study addresses these gaps by proposing a hybrid deep learning model optimized using a novel WWPA-GWO algorithm designed to improve predictive accuracy, reduce computational cost, and ensure robust statistical validation for early CKD prediction.

## Materials and methods

### Data collection

Chronic kidney disease data were culled from the UCI Repository at the University of California, Irvine. The data collection contains 400 patient records, some of which are incomplete. One class feature represents the projected occurrence of chronic renal failure, while the other 24 clinical traits are associated with the prognosis of chronic kidney disease. In the diagnostic for anticipated features, you’ll see the values “ckd” and “notckd.” There are 250 “ckd” values (62.5% of the total) and 150 “notckd” values (37.5%) in the dataset.

The dataset utilized in this study contains 24 features that encompass demographic, clinical, and laboratory attributes relevant to the diagnosis of CKD. These features collectively capture patient-specific indicators, including vital signs, urinary and blood biomarkers, and comorbid conditions, making the dataset rich in multidimensional patterns essential for reliable prediction.

The target variable is the class label, which categorizes patients as either ckd (positive diagnosis) or notckd (negative diagnosis). The remaining 23 attributes serve as input variables for the classification models. These features, summarized in Table 2, include both numerical and categorical variables such as blood pressure, blood glucose levels, red blood cell counts, and presence of comorbidities like diabetes or hypertension. This comprehensive feature space enables the practical training of machine learning models to capture the complex, non-linear relationships underlying CKD pathology.

**Table 2:** Summary of features used in the CKD dataset.

Feature name	Description
age	Patient age (years)
bp	Blood pressure (mm/Hg)
sg	Specific gravity of urine
al	Albumin level in urine
su	Sugar level in urine
rbc	Red blood cells (normal/abnormal)
pc	Pus cell (normal/abnormal)
pcc	Pus cell clumps (present/not present)
ba	Bacteria (present/not present)
bgr	Blood glucose random (mg/dl)
bu	Blood urea (mg/dl)
sc	Serum creatinine (mg/dl)
sod	Sodium (mEq/L)
pot	Potassium (mEq/L)
hemo	Hemoglobin (g/dl)
pcv	Packed cell volume (%)
wc	White blood cell count (cells/cu mm)
rc	Red blood cell count (millions/cu mm)
htn	Hypertension (yes/no)
dm	Diabetes mellitus (yes/no)
cad	Coronary artery disease (yes/no)
appet	Appetite (good/poor)
pe	Pedal edema (yes/no)
ane	Anemia (yes/no)
class	Target class (ckd / notckd)

To ensure a balanced distribution of classes, data augmentation techniques, such as Synthetic Minority Over-sampling Technique (SMOTE), were applied to the minority class, “notckd.” This was done to avoid the issue of class imbalance that could impact the performance of the predictive model.

**Fig. 1:**
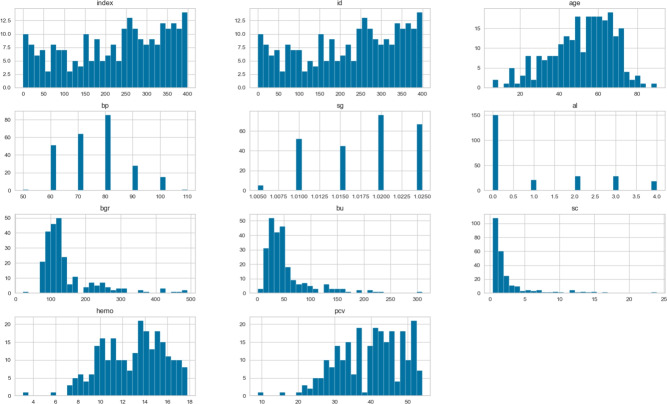
Histograms of numerical variables in the CKD dataset.

Figure 1 displays the Histogram of numerical variables from a CKD dataset. For “age,” “hemoglobin,” and “pcv,” key observations indicate a normal distribution. Histograms are crucial in understanding the distribution of each feature in the dataset, helping to identify potential outliers, skewness, or patterns that could affect the performance of machine learning models. Additionally, these visualizations can offer insights into the feature scaling requirements for the data.

### Data preprocessing

The data was normalized and validated, missing values were estimated, and outliers were removed during preparation. It is possible for an incomplete or incorrect set of measures to be used in a patient’s evaluation. Additionally, the dataset is incomplete, as it lacks information for all but 158 instances. Ignoring records is the easiest method for dealing with missing values; however, this is impractical except for very tiny data sets. During data preparation, the dataset is checked for missing attribute values. Mean imputation was used to provide an educated estimate for the missing numeric attributes. The mode approach was employed to impute missing nominal feature values. Category values must be encoded into numbers to feed the dataset into a machine-learning model. Categories such as “no” and “yes” are represented by the binary numbers “0” and “1,” respectively.

The selected method of imputation, such as mean and mode imputation, is crucial in preserving the integrity of the dataset. Other developmental imputation methods, such as K-Nearest Neighbors imputation or multiple imputation, could be further explored in future work to enhance data accuracy.

On the other hand, data transformation is applied to ensure that no single variable has an outsized impact on the final results. If the unit of measure is not specified, then learning algorithms always interpret greater values as higher and smaller ones as lower. Its values are often transformed to prepare a dataset for subsequent analysis^42^. To enhance the precision of machine learning models, this study employs a normalization method to refine the underlying data. From a negative one to a positive one, it transforms data. The transformed information has a mean of zero and a standard deviation of one. The formula for standardization is applied as follows:

1 $$\begin{aligned} w = \frac{(x - \hat{x})}{\sigma } \end{aligned}$$

where, the observed value is denoted by x, the standard deviation is denoted by $\sigma$, the standardized score is denoted by *w*, and the mean is denoted by $\hat{x}$.

Apart from normalization, scaling methods (such as Min-Max Scaling) of the features could be investigated to reduce convergence times for specific ML models, especially for deep learning models. Scaling ensures an equal contribution of all features to the model’s predictions, eliminating feature dominance due to its extensive range.

Points in the data that don’t fit in with the rest are called outliers. An outlier result may be due to a measurement error or random chance. An outlier can skew the results of a machine learning algorithm’s training phase. The outcomes include longer training times, decreased model accuracy, and worse performance. When cleaning data for a learning system, the authors of this study employ an IQR-based technique^42^.

It is here that methods such as the Z-score or IQR method become crucial in improving model accuracy. It is also beneficial for future researchers to consider more advanced techniques, such as isolation forests or one-class SVMs, to identify and manage outliers more efficiently, particularly in high-dimensional medical data.

Although the primary goal of this research is to classify patients into either ckd or notckd, the underlying predictive task was approached through a regression framework. This design enables the neural network to generate a continuous output score that reflects the likelihood of CKD presence. Such a probabilistic interpretation offers greater flexibility, especially in medical contexts where decision thresholds may vary depending on the patient’s risk profile. After the regression model generates a score between 0 and 1, a threshold (commonly 0.5) is applied to convert the output into a binary classification. This strategy not only enables the use of traditional classification metrics, such as accuracy, Sensitivity, and F1-Score, but also supports evaluation using regression-based metrics, including MSE, RMSE, and R-squared, providing a more comprehensive assessment of model performance.

Despite its advantages, applying SMOTE introduces the potential risk of overfitting, as it synthetically generates new samples that may closely resemble existing minority class instances. Overfitting occurs when the model memorizes training data-including the synthetic examples-instead of learning generalizable patterns. To mitigate this issue, this study incorporates multiple regularization strategies, including dropout layers within the neural network, early stopping during training, and rigorous cross-validation. These mechanisms collectively reduce the risk of overfitting and ensure that the model maintains robust generalization capability when evaluated on unseen test data.

### Feature selection

Recursive structure elimination (RFE) is a recursive process that recursively removes features, constructing a model based on eliminating other features^43^. It employs a greedy search algorithm to identify the subset of features that is most effective in achieving its goals. Utilize the model’s accuracy to ascertain which features best predict a feature. It generates models iteratively, evaluating each feature at each stage of development to determine if it is an improvement or a regression. Afterward, the features are organized into categories according to the order in which they were eliminated. If the data set comprises N functions, recursive feature elimination will eagerly seek a combination of 2*N* features in the worst-case scenario. This is because *N* is the number of functions in the data set.

In addition to RFE, alternative feature selection methods include *L1* regularization (Lasso) and tree-based methods, such as Random Forest feature importance, which can enhance the robustness of the feature selection process. These approaches may provide new insights into the importance of distinguishing the most predictive features.

### Regression model

The regression model adopted for predicting CKD is a deep neural network. The suggested model comprises 12 layers: an input layer, followed by five dense layers, five dropout layers, and an output dense classifier layer. Each thick layer is directly connected within this design using a feed-forward mechanism. The layer is constructed so that the outputs of its activation maps are passed on as input to all subsequent layers. This model’s dropout layer is situated between two thick layers, with drop rates of 0.5, 0.4, 0.3, 0.2, and 0.1, respectively. The CNN model has several hyperparameters that require optimization to function correctly. Selecting the ideal hyperparameters involves experimentation; nonetheless, it is a laborious, time-consuming, and complex process. During the training phase, the Adam^44^ optimizer implements hyperparameters with reduced parameter sizes. Adam determines individual learning rates for various hyperparameter grades via adaptive assessment. These grades range from first to second-order gradients. Adam is a more time and resource-effective algorithm than stochastic gradient optimization (SGD)^45^. It requires a small amount of both learning time and memory to master. The proper activation function of a CNN contributes to an improvement in classification performance. Sigmoid, tan, Rectified Linear Unit (ReLU)^46^, Exponential Linear Unit (ELU)^47^, and Self-Normalized Linear Unit (SELU)^48^ are the typical activation functions for neural networks. In this study, many activation functions were applied to the CKD data set, and the results were compared to see which performed best across all models. Apart from DNN, other regression models, such as Support Vector Regression (SVR) or Random Forest Regression, can be applied to evaluate their performance in comparison to DNN. A possible contribution to the prediction accuracy of CKD by hybrid models using these regression techniques with ensemble learning techniques may also be promising.

### Waterwheel optimization algorithm

The Waterwheel plant (Aldrovanda vesiculosa) features fascinating carnivorous traps positioned on broad petioles, resembling miniature, translucent versions of Venus flytraps, measuring approximately 1/12 inch. These remarkable structures are protected by a ring of bristle-like hairs that prevent accidental damage from contact with other aquatic vegetation. Each trap’s perimeter contains multiple hook-shaped teeth that interlace securely when capturing prey, similar to the mechanism found in Venus flytraps. The interior houses approximately forty sensitive trigger hairs-significantly more than the 6-8 found in Venus flytraps, which activate the rapid closure mechanism upon single or multiple stimulations. Beyond these trigger structures, the plant possesses specialized acid-secreting glands that facilitate the digestion of its prey. Once captured, victims become trapped by the interlocking teeth and mucus sealant, which creates a watertight enclosure that forces the prey toward the trap’s base, near the hinge joint. The digestive process expels most of the water content while breaking down the trapped organism. Each Aldrovanda trap maintains functionality for two to four capture cycles before requiring replacement, paralleling the lifecycle of Venus flytrap mechanisms^49^.

These aquatic traps rank among nature’s most rapid and efficient carnivorous mechanisms, achieving closure within milliseconds after the activation of their trigger hairs. This lightning-fast response represents a crucial evolutionary adaptation, enabling the plant to capture swift-moving aquatic prey before it can escape. The closure mechanism relies on hydraulic pressure dynamics combined with rapid cellular expansion, achieved through precise manipulation of turgor pressure within the trap’s cellular walls.

The Waterwheel Optimization Algorithm (WWPA) draws inspiration from these highly effective, rapid, and selective capturing strategies, translating them into computational optimization techniques. By emulating the plant’s ability to identify and secure high-value targets while discarding less promising alternatives, WWPA offers a novel and powerful approach to solving complex optimization challenges, particularly excelling in high-dimensional problems with noisy objective functions.

#### Initialization

WWPA operates as a population-based metaheuristic that iteratively improves solutions through the collective search capabilities of its population members within the solution space. Each waterwheel within the WWPA population represents a potential solution characterized by its position in the search space, with parameter values corresponding to problem variables. Mathematically, each waterwheel can be expressed as a solution vector, with the entire population forming the complete solution set represented by Eq. 2. The algorithm initializes waterwheel positions randomly throughout the search space using Eq. 3.

2 $$\begin{aligned} & P = \begin{bmatrix} P_1 \\ \vdots \\ P_i \\ \vdots \\ P_N \end{bmatrix} = \begin{bmatrix} p_{1,1} & \cdots & p_{1,j} & \cdots & p_{1,m} \\ \vdots & \ddots & \vdots & \vdots & \vdots \\ p_{i,1} & \cdots & p_{i,j} & \cdots & p_{i,m} \\ \vdots & \vdots & \vdots & \ddots & \vdots \\ p_{N,1} & \cdots & p_{N,j} & \cdots & p_{N,M} \end{bmatrix}\end{aligned}$$

3 $$\begin{aligned} & p_{i,j} = lb_j + r_{i,j}. (ub_j - lb_j), i = 1, 2,..., N, j= 1, 2,..., m \end{aligned}$$

Denoting the number of water wheels, and the number of variables by *N* and *m*, respectively, $r_{i,j}$ is a random number[0, 1],$lb_j$ and $ub_j$ are the lower bound and upper bound of the j-th problem variable, and *P* are populations of locations of water wheel. *Pi* – i- th waterwheel (candidate solution), $p_{i,j}$- its j-th dimension (problem variable).

Since each waterwheel corresponds to a unique solution, we can evaluate the objective function for every individual in the population. The objective function values can be efficiently represented using the vector format shown in Eq. 4.

4 $$\begin{aligned} F = \begin{bmatrix} F_1 \ \vdots \ F_i \ \vdots \ F_N \end{bmatrix} = \begin{bmatrix} F(X_1) \ \vdots \ F(X_i) \ \vdots \ F(X_N) \end{bmatrix} \end{aligned}$$

The vector *F* contains all objective function evaluations, with $F_i$ representing the fitness value for the i-th waterwheel. These objective function evaluations serve as the primary criterion for solution ranking, where the optimal candidate solution corresponds to the highest objective function value. In contrast, the poorest solution exhibits the lowest value. The best solution evolves dynamically as waterwheels navigate the search space with varying velocities across different iterations.

#### Phase 1: Prey Detection and Hunting Behavior (Exploration)

Waterwheels demonstrate exceptional predatory capabilities through their acute sensory mechanisms, enabling them to detect and track potential prey with excellent efficiency. Upon detecting nearby insects, the waterwheel initiates an aggressive pursuit sequence, systematically locating, attacking, and capturing the target. WWPA models this behavioral pattern in its initial population update phase, simulating the waterwheel’s attack strategy against insect colonies and the subsequent positional adjustments within the search space. This modeling approach enhances WWPA’s exploratory capabilities, improving its ability to identify promising regions while avoiding local optima traps. The algorithm calculates new waterwheel positions during prey approach using the following equations, where position updates are accepted only if they result in improved objective function values.

5 $$\begin{aligned} & \overrightarrow{W} = \overrightarrow{r}_1. (\overrightarrow{P}(t) + 2K)\end{aligned}$$

6 $$\begin{aligned} & \overrightarrow{P}(t+1) = \overrightarrow{P}(t) + \overrightarrow{W}. (2K + \overrightarrow{r}_2) \end{aligned}$$

When solutions fail to improve over three consecutive iterations, the algorithm applies the following position update mechanism to maintain search diversity:

7 $$\begin{aligned} \overrightarrow{P}(t+1) = Gaussian(\mu _P, \sigma ) + \overrightarrow{r}_1 \left( \frac{\overrightarrow{P}(t) + 2K}{\overrightarrow{W}} \right) \end{aligned}$$

where $\overrightarrow{r}_1$ and $\overrightarrow{r}_1$ are random variables with values in the range [0, 2] and [0, 1], respectively. In addition, *K* is an exponential variable with values in the range [0, 1], $\overrightarrow{W}$ is a vector that indicates the circle’s diameter in which the waterwheel plant will search for the promising areas.

#### Phase 2: Prey transport to digestive chamber (Exploitation)

Following successful prey capture, the waterwheel transports the captured insect into its specialized digestive tube through a tightly controlled process. WWPA’s second phase emulates this behavior, focusing on intensifying the search around promising areas that have already been discovered. This exploitation mechanism enhances local search capabilities by attracting solutions toward the neighborhood of high-quality candidates, creating refined positional adjustments within the search space. The algorithm generates new random positions representing optimal feeding locations for each waterwheel, implementing position updates only when improvements to the objective function are achieved.

8 $$\begin{aligned} & \overrightarrow{W} = \overrightarrow{r}3. (K \overrightarrow{P}{best}(t) + r_3 \overrightarrow{P}(t))\end{aligned}$$

9 $$\begin{aligned} & \overrightarrow{P}(t+1) = \overrightarrow{P}(t) + K \overrightarrow{W} \end{aligned}$$

where $\overrightarrow{r}_3$ is a random variable with values in the range [0, 2], $\overrightarrow{P}(t)$ is the current solution at iteration *t*, and $\overrightarrow{P}_{best}$ is the best solution.

Similar to the exploration phase, the algorithm applies mutation when solutions stagnate for three iterations, preventing local minima entrapment:

10 $$\begin{aligned} \overrightarrow{P}(t+1) = (\overrightarrow{r}_1 + K) \sin {\left( \frac{F}{C} \theta \right) } \end{aligned}$$

The variables *F* and *C* are random parameters within $[-5, 5]$. The parameter *K* decreases exponentially throughout the optimization process according to:

11 $$\begin{aligned} K = \left( 1 + \frac{2 * t^2}{T_{max}} +F \right) \end{aligned}$$

### Grey wolf optimization algorithm

The gray wolf optimizer recreates the behaviors of wolves as they hunt for their prey by simulating their motions. Wolves are social animals that live in groups called packs, which can range in size from five to twelve members. Four distinct types of wolves comprise a single pack: alpha, beta, delta, and omega. The choices that are made in each pack are made by the alpha wolf. The beta wolves provide assistance to the alpha wolves in decision-making. The wolves of the delta pack are submissive to the alpha and beta packs. The omega wolves are the most submissive of the pack. The alpha ($\overrightarrow{L}_\alpha$) solution is considered to be the optimal one according to mathematical standards; the beta ($\overrightarrow{L}_\beta$) and delta ($\overrightarrow{L}_\delta$) solutions, on the other hand, take second and third place, respectively. Other potential solutions are denoted with the omega symbol ($\overrightarrow{L}_\omega$). As seen in Eqs. 12, 13, 14, and 15, the alpha, beta, and delta wolves serve as guides for the other wolves as they pursue and ultimately capture their prey.

12 $$\begin{aligned} & \overrightarrow{L}(t+1) = \overrightarrow{L}_p(t) - \overrightarrow{A}.\overrightarrow{D}\end{aligned}$$

13 $$\begin{aligned} & \overrightarrow{D} = | \overrightarrow{C}. \overrightarrow{L}_p(t) - \overrightarrow{L}(t) \end{aligned}$$

If *t* is the current iteration, $\overrightarrow{A}$ and $\overrightarrow{C}$ are vectors representing the coefficients, $\overrightarrow{L}_p$ is the location of the prey, and G stands for the position of the wolf. The $\overrightarrow{A}$ and $\overrightarrow{C}$ vectors may be calculated as follows:

14 $$\begin{aligned} & \overrightarrow{A} = 2 \overrightarrow{a}. \overrightarrow{r}_1 - \overrightarrow{a}\end{aligned}$$

15 $$\begin{aligned} & \overrightarrow{C} = 2. \overrightarrow{r}_2 \end{aligned}$$

Where the components of $\overrightarrow{a}$ are decreasing linearly from 2 to 0 during the iterations, and the values of vectors $\overrightarrow{r}_1$ and $\overrightarrow{r}_2$ are random in the range [0, 1]. The value of the parameter $\overrightarrow{a}$ is adjusted, and it is used to maintain a healthy equilibrium between the exploration and exploitation processes^50^. The values of $\overrightarrow{a}$ are found by computing them according to the following equation:

16 $$\begin{aligned} \overrightarrow{a} = 2 - t. \frac{2}{M_t} \end{aligned}$$

Where $M_t$ refers to the total number of possible iterations that the optimizer has access to.

As $\overrightarrow{a}$ decreases over time, the optimizer shifts from an exploration phase (at the beginning of the iterations) to an exploitation phase (as it gets closer to an optimal solution). This shift ensures that the optimization process is both robust and adaptive, enabling it to avoid premature convergence.

The top three solutions, $\overrightarrow{L}_\alpha$, $\overrightarrow{L}_\beta$, and $\overrightarrow{L}_\delta$, direct other individuals to modify their locations so that they are closer to the predicted location of the prey. Equations 17 and 18 show the process of updating the wolves’ positions.

17 $$\begin{aligned} \begin{aligned}&\overrightarrow{D}_\delta = |\overrightarrow{C}_3. \overrightarrow{L}_\delta - \overrightarrow{L}| \\&\overrightarrow{D}_\beta = |\overrightarrow{C}_2. \overrightarrow{L}_\beta - \overrightarrow{L}| \\&\overrightarrow{D}_\alpha = |\overrightarrow{C}_1. \overrightarrow{L}_\alpha - \overrightarrow{L}| \\&\overrightarrow{L}_3 = |\overrightarrow{C}_\delta - \overrightarrow{A}_3. \overrightarrow{D}_\delta | \\&\overrightarrow{L}_2 = |\overrightarrow{C}_\beta - \overrightarrow{A}_2. \overrightarrow{D}_\beta | \\&\overrightarrow{L}_1 = |\overrightarrow{C}_\alpha - \overrightarrow{A}_1. \overrightarrow{D}_\alpha | \\ \end{aligned} \end{aligned}$$

Where $\overrightarrow{A}_1$, $\overrightarrow{A}_2$, and $\overrightarrow{A}_3$ are determined using the Eq. from Eq. 15, and $\overrightarrow{C}_1$, $\overrightarrow{C}_2$, and $\overrightarrow{C}_3$ are determined using the Eq. from Eq. 15. The current locations of the population, denoted by $\overrightarrow{L}(t + 1)$, may be stated as an average of the three solutions denoted by $\overrightarrow{L}_1$, $\overrightarrow{L}_2$, and $\overrightarrow{L}_3$ derived from Eq. 17. This expression can be written as follows:

18 $$\begin{aligned} \overrightarrow{L}(t + 1) = \frac{\overrightarrow{L}_1 + \overrightarrow{L}_2 + \overrightarrow{L}_3}{3} \end{aligned}$$

This averaging process ensures that the algorithm continues to improve its search for the optimal solution by leveraging the collective strength of the three best solutions at each iteration. This approach not only accelerates convergence but also enhances the robustness of the algorithm by reducing the likelihood of being trapped in local minima.

Moreover, the algorithm’s flexibility can be further enhanced through the use of advanced strategies, such as multi-dimensional adaptation and hybrid approaches, which combine the strengths of GWO with other optimization techniques, thereby enabling better handling of more complex or multi-modal optimization landscapes.

**Fig. 2:**
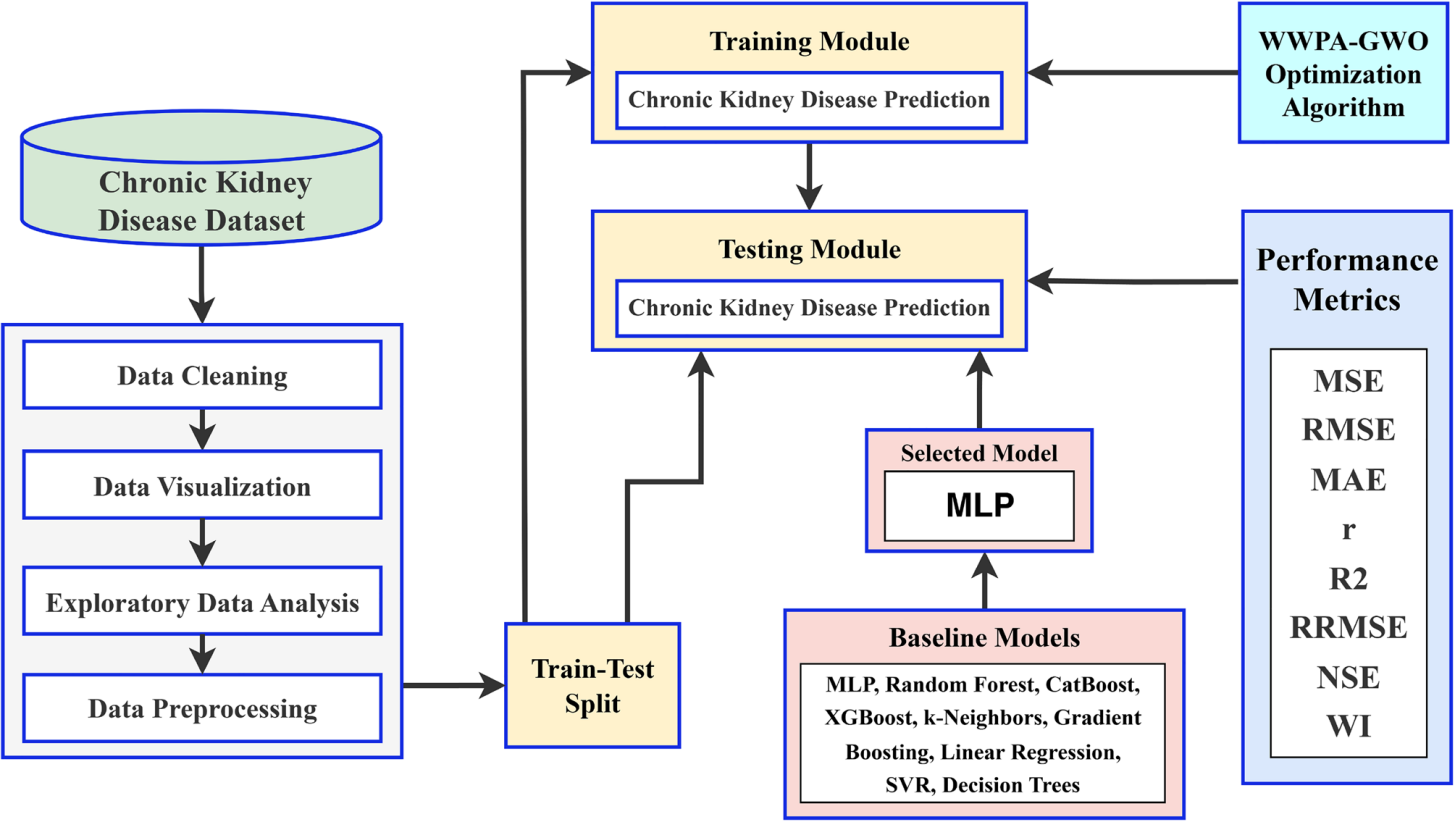
The architecture of the proposed methodology.

The basic structure of the model is illustrated in Fig. 2. The proposed optimization algorithm comprises three stages: preprocessing, model hyper-tuning, and classification. The preparation stage is critical since the data set may contain noise and redundant values. During this phase, various methodologies were employed, including those for handling missing values, encoding categorical data, data transformation, removing outliers and extreme values, and feature selection. Following the completion of the preprocessing step, the CKD dataset is partitioned into a training dataset and a testing dataset.

### The proposed hybrid WWPA-GWO optimization algorithm

The procedure of the proposed optimization algorithm is presented in Algorithm 1. The proposed optimization algorithm relies on a highly balanced exploration-exploitation strategy, a desirable characteristic of contemporary metaheuristic optimization algorithms. In WWPA-GWO, exploration is conducted to discover new and potentially superior solutions. Simultaneously, the rest is done by narrowing down on the best solutions and optimising them.

**Algorithm 1:**
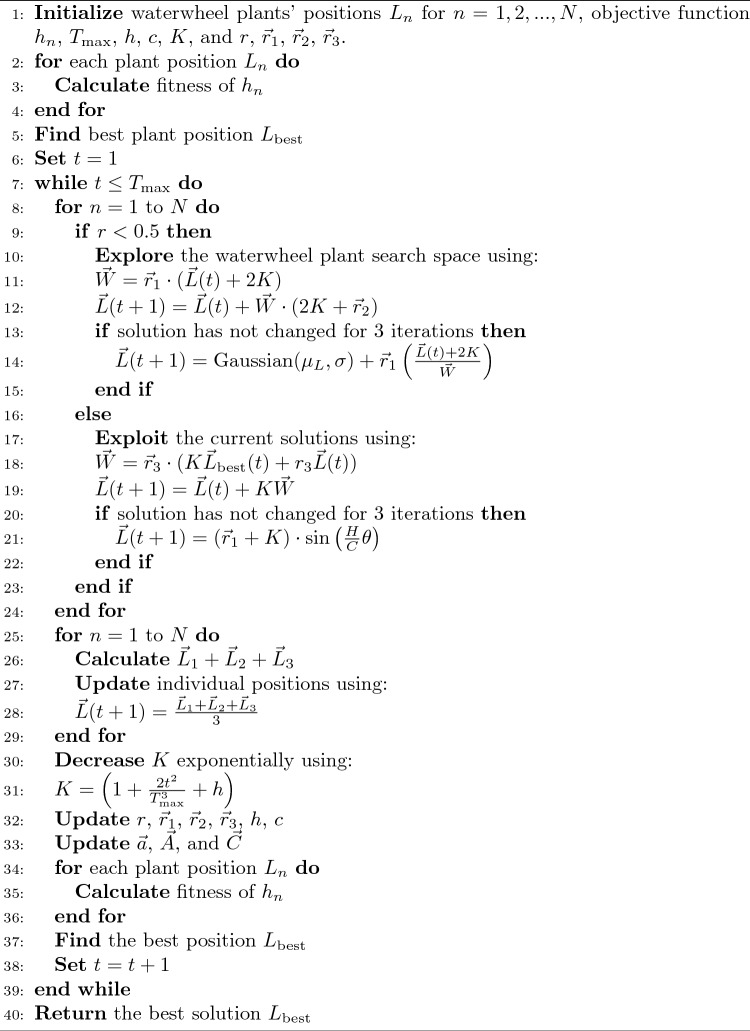
WWPA-GWO algorithm.

### Comparative metaheuristic algorithms

To evaluate the effectiveness of the proposed hybrid optimization framework for enhancing DNN training in early CKD prediction, a set of state-of-the-art metaheuristic algorithms was employed. These algorithms span multiple computational paradigms, including swarm intelligence, evolutionary computation, and bio-inspired stochastic search, and were utilized solely for optimizing the internal parameters (weights and biases) of the DNN, without applying any feature selection strategies.

The following optimization algorithms were utilized and benchmarked:

- **Waterwheel Plant Algorithm (WWPA):** Inspired by the energy dynamics of waterwheels, this algorithm simulates torque and energy flow to guide search trajectories through complex solution spaces, promoting balanced exploration and exploitation^51^.
- **Grey Wolf Optimizer (GWO):** Models the leadership hierarchy and collective hunting strategy of grey wolves, effectively balancing diversification and intensification through the guidance of alpha, beta, and delta wolves^52^.
- **Particle Swarm Optimization (PSO):** Simulates the movement of particles influenced by their own and neighbors’ previous best positions, facilitating fast convergence in continuous and discrete optimization problems^53^.
- **Whale Optimization Algorithm (WOA):** Emulates the bubble-net feeding mechanism of humpback whales, combining encircling and spiral movements for adaptive local and global search^54^.
- **Genetic Algorithm (GA):** Based on evolutionary biology, GA applies natural selection, crossover, and mutation to iteratively evolve better solutions, fostering robustness and diversity in the search process^55^.
- **Firefly Algorithm (FA):** Inspired by firefly bioluminescence and attraction behavior, FA explores the search space based on light intensity and spatial distance, enabling multiple optima discovery^56^.
- **Harris Hawks Optimization (HHO):** Mimics the cooperative and intelligent hunting style of Harris hawks, dynamically switching between exploratory and exploitative phases based on the prey’s energy^57^.
- **Fast Evolutionary Programming (FEP):** An improved evolutionary programming variant using Gaussian mutation and competitive selection to speed up convergence while maintaining solution quality^58^.
- **Stochastic Fractal Search (SFS):** Employs a diffusion and local intensification mechanism modeled on fractal growth, which enhances the exploration of high-dimensional and non-linear search spaces^59^.

By applying these algorithms to the same DNN structure, the study establishes a fair and comprehensive evaluation of their comparative performance, demonstrating the superiority of the proposed hybrid model in terms of accuracy, convergence speed, and robustness for CKD prediction.

## Evaluation metrics

To comprehensively assess the predictive accuracy and statistical robustness of the proposed hybrid optimization model for early CKD detection, a suite of standard regression-based performance metrics was employed. These metrics quantify various aspects of prediction quality, including error magnitude, variance explanation, correlation strength, and model agreement with actual observations. Their integration provides a multidimensional view of model performance, enabling fair comparison with existing approaches.

Table 3 summarizes the mathematical expressions and interpretative roles of each metric used in this study. These include both traditional measures-such as Mean Squared Error (MSE), Root Mean Squared Error (RMSE), and Mean Absolute Error (MAE)-and advanced statistical indicators like Nash–Sutcliffe Efficiency (NSE) and Willmott’s Index of Agreement (WI), which are particularly useful in model validation for medical forecasting applications.

**Table 3:** Regression performance metrics used for evaluation.

Metric	Mathematical expression and description
Mean Squared Error (MSE)	$\displaystyle \text {MSE} = \frac{1}{n} \sum _{i=1}^{n} (y_i - \hat{y}_i)^2$ Quantifies the average of the squared differences between predicted and actual values, placing a greater penalty on larger errors.
Root Mean Squared Error (RMSE)	$\displaystyle \text {RMSE} = \sqrt{\frac{1}{n} \sum _{i=1}^{n} (y_i - \hat{y}_i)^2}$ Provides an interpretable error measure in the same units as the output variable, reflecting the standard deviation of prediction errors.
Mean Absolute Error (MAE)	$\displaystyle \text {MAE} = \frac{1}{n} \sum _{i=1}^{n} |y_i - \hat{y}_i|$ Represents the average magnitude of prediction errors, offering a more robust metric against outliers.
Mean Bias Error (MBE)	$\displaystyle \text {MBE} = \frac{1}{n} \sum _{i=1}^{n} (y_i - \hat{y}_i)$ Indicates the average bias in predictions, identifying consistent under- or overestimation.
Pearson Correlation Coefficient (r)	$\displaystyle r = \frac{\sum (y_i - \bar{y})(\hat{y}_i - \bar{\hat{y}})}{\sqrt{\sum (y_i - \bar{y})^2 \sum (\hat{y}_i - \bar{\hat{y}})^2}}$ Measures the linear correlation between actual and predicted outputs; a higher value suggests stronger agreement.
Coefficient of Determination (R^2^)	$\displaystyle R^2 = 1 - \frac{\sum (y_i - \hat{y}_i)^2}{\sum (y_i - \bar{y})^2}$ Indicates the proportion of variance explained by the model; closer to 1 implies better predictive performance.
Relative Root Mean Squared Error (RRMSE)	$\displaystyle \text {RRMSE} = \frac{\text {RMSE}}{\bar{y}}$ Normalizes RMSE by the mean of actual values, enabling comparison across datasets or models.
Nash–Sutcliffe Efficiency (NSE)	$\displaystyle \text {NSE} = 1 - \frac{\sum (y_i - \hat{y}_i)^2}{\sum (y_i - \bar{y})^2}$ Assesses predictive skill by comparing model errors to the variability of the observed data; higher values imply better performance.
Willmott’s Index of Agreement (WI)	$\displaystyle \text {WI} = 1 - \frac{\sum (y_i - \hat{y}_i)^2}{\sum \left( | \hat{y}_i - \bar{y} | + | y_i - \bar{y} | \right) ^2}$ Measures the degree of error relative to observed variance, with values closer to 1 indicating high predictive accuracy.

The collective use of these evaluation metrics enables a nuanced understanding of the model’s generalization capability, robustness, and practical applicability for clinical decision-making.

## Experimental results

In this section, the results obtained using the proposed methodology are discussed. The adopted dataset is first preprocessed, and the proposed optimization algorithm is then trained, and its parameters are optimized using the proposed algorithm. Then, a promising model is selected and used for further experimentation. The adopted model is optimized using the suggested optimization algorithm, and the results of the chronic disease prediction for this model are presented and discussed in this section.

### Experimental setup

In this study, all algorithms were evaluated under identical experimental conditions to ensure the fairness of comparison. The details of the parameter configurations are provided in Table 4. Each optimizer was executed with its respective parameter ranges, ensuring consistency across the evaluation process. The population size and number of iterations were kept constant for all methods. In contrast, algorithm-specific parameters such as random numbers, learning factors, and mutation or crossover probabilities were set according to their typical configurations. This uniform setup guarantees that performance differences arise from the inherent characteristics of the algorithms rather than unequal parameter tuning.

**Table 4:** Configuration parameters of the proposed and comparative algorithms.

Algorithm	Parameter	Value/Range
WWPA-GWO	$r_2, r_3, r_4$	[0,1]
WWPA	$r_2, r_3, r_4$	[0,1]
GWO	$r_1, r_2$	(0,1)
PSO	$w=0.68$; $c_1, c_2=0.5$	–
WOA	$b=1$; $p \in (0,1)$	–
GA	$P_c=0.8$; $P_m=0.2$; $gap=0.9$	–
FA	$\alpha =0.25$; $\gamma =1.0$; $r \in [0,1]$	–
SFS	*r*	0.1

To further enhance reproducibility and transparency, Table 5 summarizes the key hyperparameters of the DNN used in this study. These include the architecture configuration, activation functions, learning rate, dropout strategy, batch size, and optimization algorithm. Such detailed reporting ensures that other researchers can accurately reproduce the model setup.

**Table 5:** Summary of deep neural network (DNN) hyperparameters.

Hyperparameter	Description / Value
Number of Layers	12 (1 input, 5 dense, 5 dropout, 1 output)
Neurons per Dense Layer	[128, 64, 32, 16, 8]
Activation Functions	ReLU for hidden layers; Sigmoid for output layer
Dropout Rates	0.5, 0.4, 0.3, 0.2, 0.1 (between dense layers)
Optimizer	Adam optimizer^42^
Learning Rate	0.001 (adaptively adjusted via WWPA-GWO)
Batch Size	32
Number of Epochs	100 (with early stopping)
Loss Function	Mean Squared Error (MSE)
Evaluation Metrics	MSE, RMSE, MAE, R^2^, NSE, WI
Regularization Techniques	Dropout layers, early stopping, and cross-validation

By providing this summary, the experimental design becomes fully transparent, enabling reproducibility and allowing future studies to benchmark or extend the proposed WWPA–GWO-optimized DNN under comparable configurations.

### Regression models evaluation

The results tabulated in Table 6 give an exhaustive overview of the effectiveness of various regression models in predicting CKD. Such an exploration provides valuable insights into the suitability of these models for medical diagnosis and prognosis. Firstly, it is essential to assess the predicted accuracy, and measures such as MSE, RMSE, and MAE are helpful for this task. Lower ones represent better prediction performance for all criteria. The Multilayer Perceptron model achieved better performance compared to other models under consideration, with the lowest values of MSE=0.00177, RMSE=0.04202, and MAE=0.01002, validating the fact that the MLP model’s output is most comparable to the actual values.

**Table 6:** Prediction results achieved using various regression models.

Regression Model	MSE	RMSE	MAE	MBE	r	R^2^	RRMSE	NSE	WI
MLPRegressor	0.002	0.042	0.010	0.007	0.867	0.879	7.1	0.887	0.864
RandomForestRegressor	0.011	0.104	0.012	0.011	0.850	0.863	9.1	0.865	0.855
ExtraTreesRegressor	0.013	0.115	0.014	0.012	0.847	0.840	9.3	0.861	0.856
CatBoost	0.015	0.122	0.016	0.021	0.835	0.838	9.5	0.850	0.804
XGBoost	0.028	0.166	0.044	0.046	0.805	0.808	9.7	0.828	0.782
Pipeline	0.029	0.172	0.048	0.049	0.810	0.832	10.6	0.824	0.774
KNeighborsRegressor	0.044	0.211	0.053	0.054	0.797	0.806	11.0	0.814	0.761
GradientBoostingRegressor	0.055	0.234	0.060	0.063	0.780	0.802	12.2	0.813	0.751
LinearRegression	0.065	0.255	0.078	0.098	0.777	0.806	13.4	0.792	0.751
SVR	0.083	0.287	0.089	0.120	0.767	0.776	13.7	0.792	0.738
DecisionTreeRegressor	0.121	0.348	0.103	0.158	0.767	0.776	14.1	0.790	0.728

**Fig. 3:**
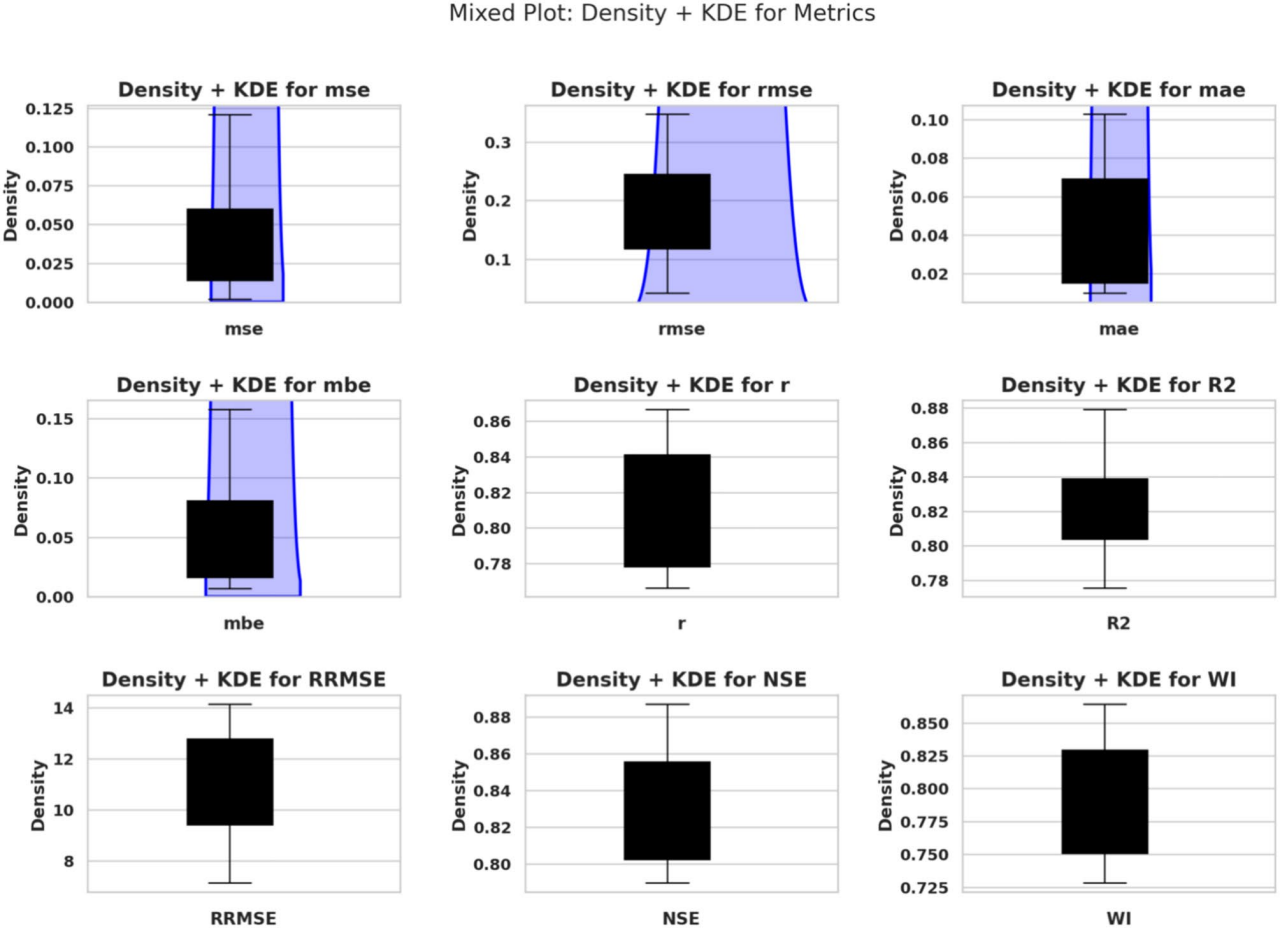
Mixed plot (Density + KDE) for the distribution of performance metrics: MSE, RMSE, MAE, MBE, *r*, $R^2$, RRMSE, NSE, and WI.

The distributions of MSE, RMSE, and MAE appear in the first three subplots of Fig. 3. The fourth subplot in this analysis displays MBE values, indicating systematic prediction biases present in the models.

**Fig. 4:**
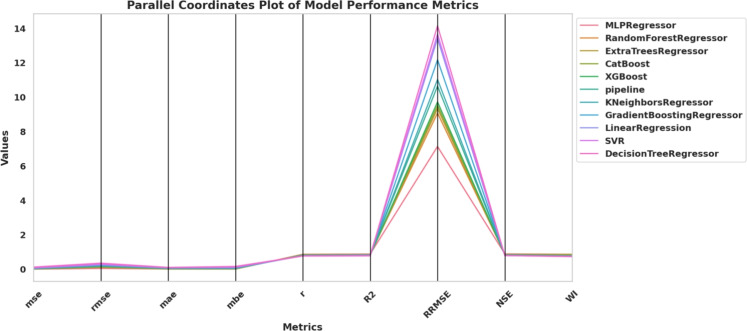
Parallel coordinates plot showing the performance of baseline regression models across various evaluation metrics.

The simultaneous display of evaluation metric behavior across multiple regression models is illustrated in Fig. 4 using a parallel coordinates plot. A set of evaluation metrics that includes MSE, RMSE, MAE, MBE, *r*, $R^2$, RRMSE, NSE and WI appears in this figure.

**Fig. 5:**
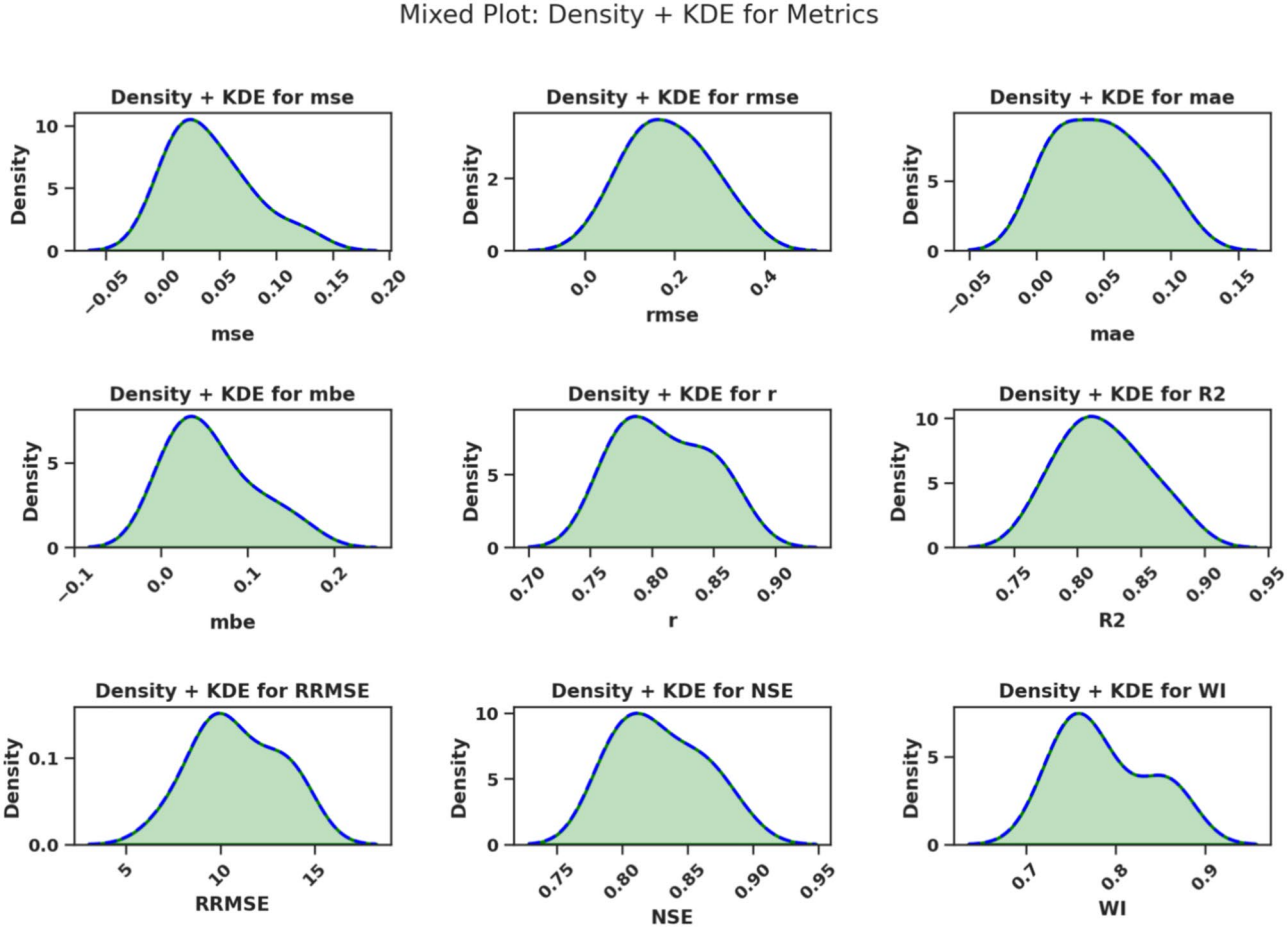
Density and Kernel Density Estimation (KDE) plots of the performance metrics: MSE, RMSE, MAE, MBE, *r*, $R^2$, RRMSE, NSE, and WI.

MSE distributions appear with RMSE and MAE in the first row of Fig. 5. The second row includes MBE, *r*, and $R^2$. The third row provides density distributions of RRMSE, NSE, and WI. The graphical representations offer viewers with a clearer understanding of how each metric behaves and varies across models.

### Optimization algorithm results

The findings presented in Table 7 provide a comprehensive perspective on the predictive performance of various optimization methods when applied to the goal of improving the prediction of CKD. The proposed optimization algorithm is shown to outperform the other nine optimization methods. These models have been thoroughly examined using a variety of important performance measures, which have shed light on their efficacy in producing accurate predictions and assisting medical diagnosis. Let’s start by concentrating on MSE and RMSE. These metrics provide an essential measure for predicting accuracy, with lower values indicating better performance. The WWPA-GWO model stands out from its competitors because it has the lowest mean squared error (MSE) $3.06 \times 10^{-6}$ and root mean squared error (RMSE) of 0.00175.

**Table 7:** The prediction results achieved using the proposed optimization algorithm based on various optimization algorithms.

Models	MSE	RMSE	MAE	MBE	r	R^2^	RRMSE	NSE	WI
WWPA-GWO	3.06E−06	0.00175	5.09E−05	5.97E−05	0.96752	0.97305	0.48391	0.96287	0.96001
WWPA	3.81E−05	0.00617	0.00104	0.00040	0.94040	0.95026	0.67836	0.95140	0.94815
GWO	9.29E−05	0.00964	0.00105	0.00046	0.93883	0.94436	0.76997	0.94772	0.94076
PSO	1.10E−04	0.01047	0.00105	0.00052	0.93721	0.94274	0.85815	0.94576	0.93815
WOA	1.66E−04	0.01288	0.00107	0.00061	0.92504	0.94189	0.92789	0.94019	0.93618
GA	1.76E−04	0.01327	0.00109	0.00066	0.92415	0.93970	0.98173	0.93696	0.93294
FA	1.80E−04	0.01342	0.00110	0.00070	0.92333	0.93556	1.03473	0.92663	0.93458
HHO	2.49E−04	0.01579	0.00111	0.00084	0.92101	0.93141	1.18149	0.92403	0.92937
FEP	2.86E−04	0.01690	0.00112	0.00095	0.91975	0.93030	1.24544	0.92142	0.92793
SFS	3.91E−04	0.01978	0.00113	0.00096	0.91610	0.92908	1.27447	0.91938	0.92699

**Fig. 6:**
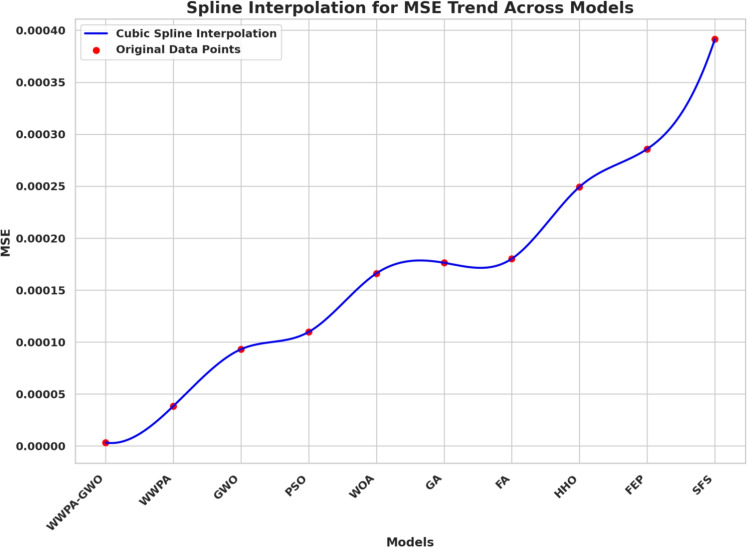
Spline interpolation of the MSE trend across the optimization algorithms.

MSE optimization trends become visible through cubic spline interpolation in Fig. 6. The precise examination of performance differences between models becomes possible due to MSE values, which smoothly advance throughout the tested models. The displays enable users to observe which optimization methods yield the lowest error rates, facilitating easier comparison of predictive outcomes.

**Fig. 7:**
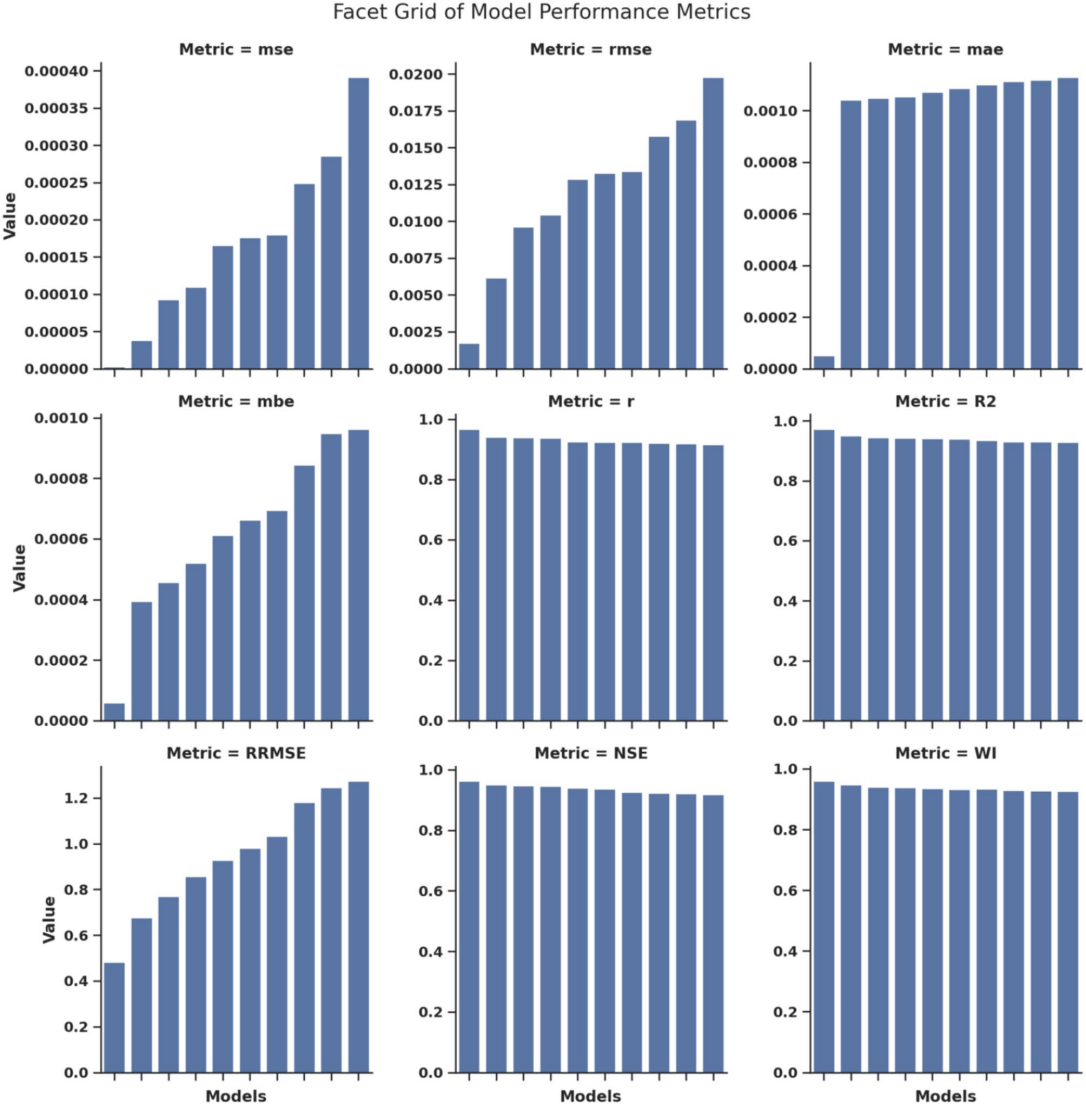
Facet grid of bar charts illustrating the performance of the optimization algorithms across multiple evaluation metrics.

The performance assessment of the optimization algorithms is presented in Fig. 7, which utilizes bar charts to facilitate easy comparison of multiple metrics. The different subplots in this figure display the quantitative performance results for MSE, RMSE, MAE, MBE, *r*, $R^2$, RRMSE, NSE, and WI. The facet grid design enables direct performance comparison of algorithms by placing them side by side, allowing users to easily see predictive variations.

When applied to the problem of CKD prediction, the findings of the given ANOVA test, presented in Table 8, provide valuable insights into the statistical significance of the suggested optimized deep network. ANOVA is a robust technique that enables us to compare the means of various groups. This allows us to determine whether there are significant differences between these groups. In this case, the “Treatment” group refers to the ten different optimization algorithms applied to the proposed optimization algorithm, whereas the “Residual” group is responsible for accounting for the variability within each of these models. The “Total” numbers represent the entire range of variation that may be found in the dataset.

**Table 8:** ANOVA test results for comparing the performance of different optimization algorithms.

ANOVA Table	SS	DF	MS	F (DFn, DFd)	*P* value
Treatment (between columns)	0.000001173	9	1.304E−07	F (9, 90) = 89.96	*P* < 0.0001
Residual (within columns)	1.304E−07	90	1.449E−09		
Total	0.000001304	99			

When applied to predicting CKD using an optimized deep network, the findings of the Wilcoxon signed-rank test reported in Table 9 provide valuable insights into the efficacy of various optimization algorithms. This non-parametric statistical test is invaluable for comparing the paired observations of various algorithms, as it helps determine whether there are significant variations in the performance of each algorithm. The number for each algorithm’s “Theoretical median” is set to 0, suggesting that, in theory, there should be no substantial difference between the algorithms’ performances. This value is set to be the same for all algorithms.

**Table 9:** Wilcoxon Signed-Rank Test Results for Optimized MLP Accuracy Scores.

Statistic	Ninja + MLP	GA + MLP	PSO + MLP	GWO + MLP	AOA + MLP	QIO + MLP
Theoretical median	0	0	0	0	0	0
Actual median	0.9894	0.9663	0.9635	0.9587	0.9535	0.9496
Number of values	10	10	10	10	10	10
Sum of signed ranks (W)	55	55	55	55	55	55
Sum of positive ranks	55	55	55	55	55	55
Sum of negative ranks	0	0	0	0	0	0
*P*-value (Two-Tailed)	0.002	0.002	0.002	0.002	0.002	0.002
Exact or estimate?	Exact	Exact	Exact	Exact	Exact	Exact
*P*-value summary	**	**	**	**	**	**
Significant ($\alpha = 0.05$)?	Yes	Yes	Yes	Yes	Yes	Yes
Discrepancy	0.9894	0.9663	0.9635	0.9587	0.9535	0.9496

**Fig. 8:**
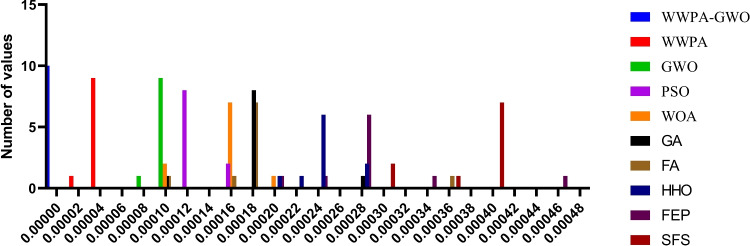
Histogram illustrating the distribution of performance metric values across the optimization algorithms.

Figure 8 shows how performance metrics from optimization algorithms are distributed through the usage of a histogram plot. This chart makes it easy to see how different evaluated methods perform because it shows the distribution of metric values. Identifying regular performance behavior becomes possible through this type of analysis, along with detecting algorithms that successfully reach target range values.

**Fig. 9:**
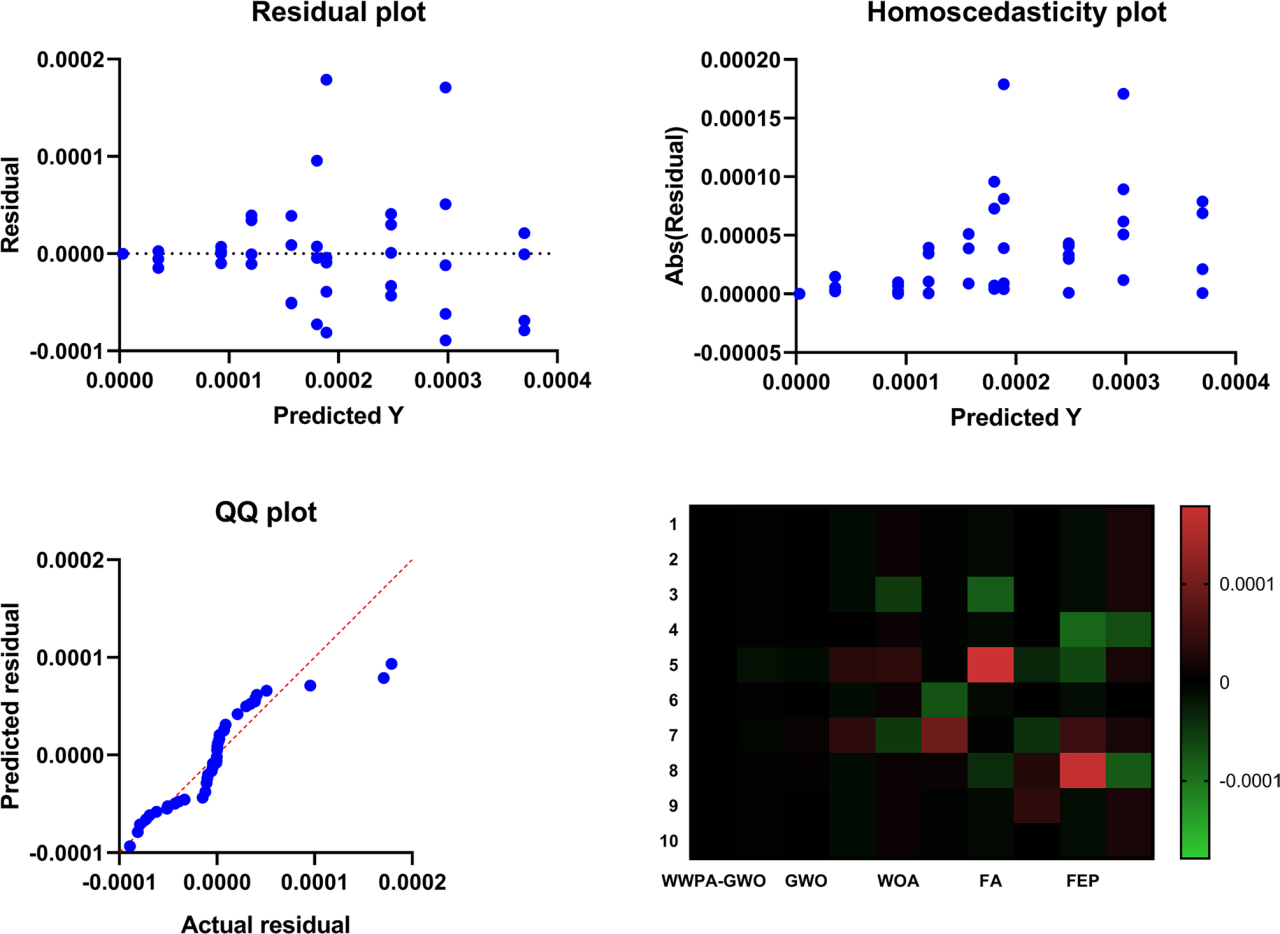
Regression diagnostic plots including residual analysis, Q-Q plot, and residual heatmap for evaluating model assumptions and fit quality.

Figure 9 reveals three subplots that show residual distributions as (1) residuals versus fitted values and (2) standardized residuals versus fitted values, along with (3) residuals versus leverage for detecting heteroscedasticity and non-linearity, and extreme points. The fourth display evaluates the normality of residuals using a Q-Q plot, which compares the actual quantiles of the residuals to theoretical distributions.

**Fig. 10:**
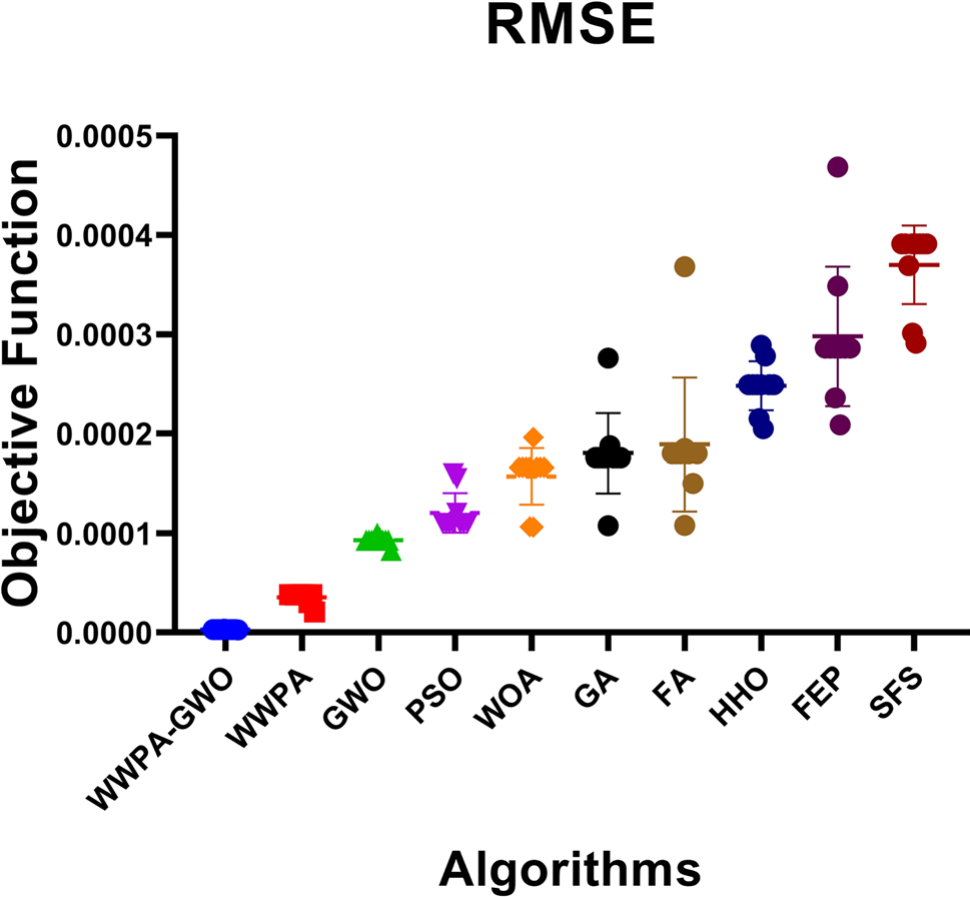
Scatter plot with error bars and distinct markers for the optimization algorithms, representing mean performance and variability across models.

The comparison of performance variability between optimization algorithms is presented in Fig. 10, which utilizes error bars with individual markers for each model. The visualization approach effectively represents both location information about performance metrics and their spread statistics, enabling users to better understand model stability.

The findings of the statistical analysis of the prediction of chronic renal disease using the proposed optimization algorithm give significant insights into the performance and features of various optimization techniques as presented in Table 10. These results shed light on various statistical features and metrics that help evaluate the models’ dependability and stability. When we first look at the column labeled “Number of values,” we see that each method was tested using the same dataset with ten different examples. Because of this consistency, it is possible to compare their respective performances accurately. The various measures of central tendency, including “Minimum,” “25% Percentile,” “Median,” “75% Percentile,” and “Maximum,” provide a comprehensive perspective on the distribution of prediction errors produced by the different algorithms.

**Table 10:** Statistical analysis results of RMSE values for the proposed optimized MLP regression model.

Metric	WWPA-GWO	WWPA	GWO	PSO	WOA
Number of values	10	10	10	10	10
Harmonic mean	0.0003	0.0006	0.0008	0.0008	0.0016
Geometric mean	0.0003	0.0006	0.0008	0.0008	0.0017
Geometric SD factor	1.127	1.054	1.066	1.039	1.312
Lower 95% CI (Geo. Mean)	0.0003	0.0006	0.0007	0.0008	0.0014
Upper 95% CI (Geo. Mean)	0.0003	0.0007	0.0008	0.0008	0.0021
Lower 95% CI (Harm. Mean)	0.0003	0.0006	0.0007	0.0008	0.0014
Upper 95% CI (Harm. Mean)	0.0003	0.0007	0.0008	0.0008	0.0021
Lower 95% CI (Quad. Mean)	0.0003	0.0006	0.0007	0.0008	0.0014
Upper 95% CI (Quad. Mean)	0.0003	0.0007	0.0008	0.0008	0.0022
Quadratic mean	0.0003	0.0006	0.0008	0.0008	0.0018
Mean	0.0003	0.0006	0.0008	0.0008	0.0018
Median	0.0003	0.0006	0.0008	0.0008	0.0017
Minimum	0.0002	0.0006	0.0006	0.0008	0.0010
Maximum	0.0003	0.0007	0.0008	0.0009	0.0030
Range	0.0001	0.0001	0.0002	0.0001	0.0020
25% Percentile	0.0003	0.0006	0.0008	0.0008	0.0016
75% Percentile	0.0003	0.0006	0.0008	0.0008	0.0018
10% Percentile	0.0002	0.0006	0.0006	0.0008	0.0010
90% Percentile	0.0003	0.0007	0.0008	0.0009	0.0029
Lower 95% CI (Mean)	0.0003	0.0006	0.0007	0.0008	0.0014
Upper 95% CI (Mean)	0.0003	0.0007	0.0008	0.0008	0.0021
Lower confidence limit	0.0003	0.0006	0.0008	0.0008	0.0015
Upper confidence limit	0.0003	0.0006	0.0008	0.0008	0.0020
Std. Deviation	0.0000	0.0000	0.0000	0.0000	0.0005
Std. Error of Mean	0.0000	0.0000	0.0000	0.0000	0.0002
Coefficient of Variation (%)	10.62	5.340	6.021	3.805	28.26
Skewness	− 2.379	0.9664	− 2.471	0.0641	1.426
Kurtosis	5.466	4.960	7.353	4.051	4.693
Actual confidence level	97.85%	97.85%	97.85%	97.85%	97.85%
Sum	0.0028	0.0063	0.0076	0.0082	0.0176

## Conclusion

Chronic Kidney Disease represents a growing global health challenge, primarily driven by diabetes and hypertension. Traditional diagnostic approaches rely heavily on Glomerular Filtration Rate measurements and other indicators of kidney dysfunction, yet early detection remains problematic in clinical practice. Since CKD involves progressive renal function deterioration and significantly increases mortality risk, timely diagnosis becomes crucial for improving patient outcomes and reducing disease-related deaths.

This research addresses the diagnostic challenge by introducing an innovative early prediction framework based on an optimized deep neural network. Our approach leverages a novel hybrid optimization algorithm that combines the strengths of the waterwheel plant algorithm with grey wolf optimization techniques, creating a powerful synergy for enhanced predictive performance.

We conducted comprehensive evaluations using multiple prediction models to validate the effectiveness of our proposed method. To demonstrate the superiority of our hybrid optimization approach, we benchmarked it against ten established optimization algorithms applied to identical deep network architectures. The comparative analysis revealed significant improvements in prediction accuracy, computational efficiency, and overall model performance.

Our multilayer perceptron model exhibited a remarkable transformation following optimization, with the mean squared error decreasing from 0.00177 to $3.06 \times 10^{-6}$, and computational time reducing to just 0.0999 seconds. These substantial improvements demonstrate both enhanced accuracy and operational efficiency.

Statistical validation through rigorous testing frameworks, including ANOVA and Wilcoxon signed-rank tests, confirmed the statistical significance of our improvements. The ANOVA results yielded a highly significant P-value of <0.0001, while the Wilcoxon signed-rank test produced a P-value of 0.002, both of which strongly support the superiority of our method over conventional approaches.

This work contributes a promising solution for early CKD detection, potentially enabling healthcare providers to intervene earlier in disease progression and ultimately improve patient outcomes in clinical practice.

Despite the promising results, this study has several limitations. First, the dataset used may not fully represent the heterogeneity of broader clinical populations, which may affect generalizability. Second, while the hybrid WWPA-GWO algorithm demonstrated superior performance, it is computationally intensive and has yet to be validated in real-time or clinical settings. Third, the model’s interpretability remains limited-a crucial factor for clinical adoption. Future work should explore the integration of explainable AI tools, larger and more diverse datasets, and real-world clinical deployment to overcome these limitations.

## Data Availability

The dataset used in this study can be found in https://archive.ics.uci.edu/dataset/336/chronic+kidney+disease
